# Microbiological profile of bacteria causing ventilator-associated pneumonia in the intensive care unit of a high-complexity hospital

**DOI:** 10.17843/rpmesp.2023.401.12377

**Published:** 2023-03-24

**Authors:** Luis Ángel Rodríguez-Chávez, Maribel Luz Esteban-Dionicio, Cristhian Renzho Elsayed Rodriguez-Mendoza

**Affiliations:** 1 Medicine faculty, Universidad Privada Antenor Orrego, Trujillo, Peru. Universidad Privada Antenor Orrego Medicine faculty Universidad Privada Antenor Orrego Trujillo Peru; 2 High complexity hospital in La Libertad “Virgen de la Puerta” Trujillo, Perú. High complexity hospital in La Libertad “Virgen de la Puerta” Trujillo Peru

Mr. Editor. Ventilator-associated pneumonia (VAP), a healthcare-associated infection, is defined as a pulmonary infection that occurs in a patient who has been on mechanical ventilation for more than 48 hours ^1^. In order to stablish diagnosis, the Peruvian Social Security (EsSalud) defines VAP as a pulmonary complication after 48 to 72 h of endotracheal intubation in patients undergoing mechanical ventilation; in addition to new or progressive infiltrates, consolidation, cavitation or pleural effusion on chest X-ray, and at least one of the following signs: purulent sputum or change in sputum characteristics, fever, increased or decreased leukocyte count, presence of cultured microorganisms in blood, or identification of a microorganism in bronchoalveolar lavage or biopsy ^2^^,^^3^. In 2012, the incidence density of VAP, according to data from the Peruvian Ministry of Health (MINSA), was 10.6 cases per 1000 days of mechanical ventilator use; the lowest incidence density was reported in 2018 with 7.56 cases per 1000 days of mechanical ventilator use. However, by 2021 the cumulative incidence rose, even above historical values, to 11.66 cases per 1000 days of mechanical ventilator use ^4^, this probably was a consequence of the COVID-19 pandemic.

Therefore, a descriptive study was conducted with the aim of identifying the cumulative incidence of VAP, as well as the most common microorganisms and their antimicrobial sensitivity profile, in the intensive care unit (ICU) of the high complexity hospital “Virgen de La Puerta” of La Libertad, Peru, during January to December 2022. The population consisted of all positive bronchial secretion cultures from patients diagnosed with VAP. Samples were processed with the VITEK system (bioMérieux Laboratory, Argentina), which is an automated system for bacterial identification and antimicrobial sensitivity study. Bacterial identification is based on the inoculation of a suspension of microorganisms on cards with certain biochemical reaction panels ^5^.

A total of 339 results were obtained from 7546 days of mechanical ventilator use (total sum of the number of days of mechanical ventilator use of each of the studied patients), which represents a cumulative incidence of 44.92 cases per 1000 days of mechanical ventilator use (much higher than the national value reported by MINSA). The most frequently isolated bacteria were *Acinetobacter baumannii* with 117 cases (35.5%), *Pseudomonas aeruginosa* with 76 cases (22.4%), *Klebsiella pneumoniae* with 49 cases (14.5%) and *Staphylococcus aureus* with 46 cases (13.6%). The remaining 15.0% were distributed among the other germs (Table 1).

**Table 1:** Absolute and percentage distribution of germs that caused mechanical ventilator-associated pneumonias in the intensive care unit of the high complexity hospital "Virgen de La Puerta" of La Libertad.

Isolated germs	n	%
*Acinetobacter baumannii complex*	117	34.5
*Pseudomona aeruginosa*	76	22.4
*Klebsiella pneumoniae*	49	14.5
*Staphylococcus aureus*	46	13.6
*Stenotrophomonas maltophilia*	13	3.8
*Escherichia coli*	10	2.9
*Enterobacter cloacae complex*	10	2.9
*Enterobacter aerogenes*	7	2.1
*Serratia marcescens*	4	1.2
*Sphingomonas paucimobilis*	2	0.6
*Citrobacter freundii*	1	0.3
*Klebsiella oxytoca*	1	0.3
*Pseudomonas fluorescens*	1	0.3
*Proteus mirabilis*	1	0.3
*Pseudomonas putida*	1	0.3
Total	339	100.0

Source: data obtained from patient antibiograms.

Antimicrobial sensitivity differed according to the type of isolated bacteria. *Acinetobacter baumannii* showed very low sensitivity to carbapenems (10.2% for meropenem and 13.0% for imipenem), quinolones (10.4% for ciprofloxacin and 13.5% for levofloxacin) and cephalosporins (0.0% for ceftriaxone, 13.1% for cefepime and 14.0% for ceftazidime), on the other hand it showed sensitivity rates above 80.0% to colistin and tigecycline (85.9% for tigecycline and 100.0% for colistin). Likewise, *Pseudomonas aeruginosa* showed very low sensitivity to carbapenems (30.6% for meropenem and 34.7% for imipenem), quinolones (50.0% for ciprofloxacin and 50.0% for levofloxacin), cephalosporins (0.0% for ceftriaxone, 48.1% for cefepime and 54.5% for ceftazidime), aminoglycosides (68.9% for amikacin, 52.8% for gentamicin) and piperacillin/tazobactam (44.9%), while having high sensitivity to colistin (100.0%). *Klebsiella pneumoniae* showed better sensitivity to carbapenems (96.7% for meropenem and 98.0% for imipenem), quinolones (72.9% for ciprofloxacin and 81.2% for levofloxacin), cephalosporins (93.9% for cefepime and 90.6% for ceftazidime), aminoglycosides (98.0% for amikacin and 88.0% for gentamicin) and piperacillin/tazobactam (96.9%). *Staphylococcus aureus* showed a 100.0% sensitivity rate to daptomycin, rifampicin, teicoplasmin and tigecycline; unfortunately, no data was available for oxacillin and vancomycin.

According to our results, it is very likely that the COVID-19 pandemic changed the frequency rates of germs causing VAP. For example, *Acinetobacter baumannii* ranked first, with 117 cases (34.5%); this is consistent with what was reported by the Cayetano Heredia National Hospital (HNCH) ^1^ during 2020, they reported that this germ was the main cause of VAP, although with higher frequency (53.0%).

*Pseudomonas aeruginosa* showed low sensitivity to cephalosporins, quinolones, amikacin and carbapenems. These data show a non-favorable sensitivity profile and are similar to those reported by the HNCH, who reported 40.0% to 50.0% sensitivity rates to carbapenems ^1^.

The present study describes the VAPs that occurred during the COVID-19 pandemic and coincides with a similar study in European ICUs after the second and third waves ^6^^,^^7^^)^ that reported that the most frequently isolated germs were *Pseudomonas aeruginosa*, *Escherichia coli*, *Klebsiella pneumoniae* and *Acinetobacter* spp. and gram-positive cocci, such as *Staphylococcus aureus*.

One of the limitations of this study was that culture samples were taken from bronchial secretion at the time of aspiration and not from bronchoalveolar lavage. Another limitation was that antibiogram results for *Staphylococcus aureus* did not include data for oxacillin and vancomycin, due to lack of supplies.

Finally, it is very possible that one of the causes of the increase in VAP with associated bacterial resistance was the indiscriminate use of antimicrobials during the COVID-19 pandemic ^8^. In this sense, the Centers for Disease Control and Prevention (CDC) reported an increase in infections by resistant germs, particularly carbapenem-resistant *Acinetobacter*, which increased by 78%; infections by multidrug-resistant *Pseudomonas aeruginosa* increased by 32%, infections by vancomycin-resistant *Enterococcus* increased by 14% and methicillin-resistant *Staphylococcus aureus* infections increased by 13% ^9^. Because of this, it was stated that the COVID-19 pandemic “set the U.S. back in its fight against bacterial resistance” ^9^. Also, the use of gloves by healthcare personnel (which affected the frequency of hand washing) may have played a role in the increase in resistant infections. Fortunately, this practice was disregarded in its entirety in 2022, so we expect the results of future studies to differ.

In conclusion, the cumulative incidence of VAP in the ICU of the “Virgen de La Puerta” hospital was 44.92 cases per 1000 days of mechanical ventilator use, and there was a change in the bacteriological and resistance profile of these infections due to the COVID-19 pandemic with a predominance of infection by germs with high antibacterial resistance.
