# Supplementary material for: Microbiological profile of bacteria causing ventilator-associated pneumonia in the intensive care unit of a high-complexity hospital
Source: Rev Peru Med Exp Salud Publica. 2023 Mar 24;40(1):115–7. doi: 10.17843/rpmesp.2023.401.12377 (PMC10953659; doi:10.17843/rpmesp.2023.401.12377)
Supplement: Translation

# Translation: Perfil microbiológico de las bacterias causantes de neumonía asociada a ventilador mecánico en la unidad de cuidados intensivos de un hospital de alta complejidad

**Authors:** Luis Ángel Rodríguez-Chávez, Maribel Luz Esteban-Dionicio, Cristhian Renzho Elsayed Rodriguez-Mendoza

**Affiliations:** 1 Facultad de Medicina, Universidad Privada Antenor Orrego, Trujillo, Perú.; 2 Hospital de Alta complejidad de La Libertad Virgen de la Puerta Trujillo, Perú.

Sr. Editor. Dentro de las infecciones asociadas al cuidado de la salud se encuentra la neumonía asociada al ventilador (NAV), definida como infección pulmonar que ocurre en un paciente que ha estado en ventilación mecánica durante más de 48 horas ^1^. Para su diagnóstico, el Seguro Social del Perú (EsSalud) lo define como complicación pulmonar después de 48 a 72 h de la intubación endotraqueal en pacientes sometidos a ventilación mecánica; además de infiltrados nuevos o progresivos, consolidación, cavitación o derrame pleural en la radiografía de tórax, y al menos uno de los siguientes signos: esputo purulento o cambio en las características del esputo, fiebre, incremento o disminución de la cuenta leucocitaria, presencia de microorganismos cultivados en sangre, o identificación de un microorganismo en lavado bronco alveolar o biopsia ^2^^,^^3^^).^ La densidad de incidencia de las NAV, según datos del Ministerio de Salud (MINSA) del Perú para el 2012 fue de 10,6 casos × 1000 días de uso de ventilador mecánico, llegando a su punto más bajo en el 2018 con 7,56 casos × 1000 días de uso de ventilador mecánico. Sin embargo, para el 2021 la incidencia acumulada subió, incluso por encima de valores históricos, a 11,66 casos × 1000 días de uso ventilador mecánico ^(^^4^, esto probablemente como consecuencia de la pandemia de la COVID-19.

Por tal razón, se realizó un estudio descriptivo con el objetivo de identificar la incidencia acumulada de la NAV, además de los microorganismos más comunes, con su perfil de sensibilidad antimicrobiana, en la unidad de cuidados intensivos (UCI) del hospital de alta complejidad «Virgen de La Puerta» de La Libertad en Perú, durante enero a diciembre del 2022. La población estuvo conformada por todos los resultados positivos de los cultivos de secreción bronquial de los pacientes con diagnóstico de NAV. Las muestras fueron procesadas con el sistema VITEK (Laboratorio bioMérieux, Argentina) que es un sistema automatizado de identificación bacteriana y estudio de la sensibilidad antimicrobiana. La identificación de las bacterias se basa en la inoculación de una suspensión de microorganismos en tarjetas con determinados paneles de reacciones bioquímicas ^5^.

Se obtuvo 339 resultados de un total de 7546 días de uso ventilador mecánico (sumatoria total del número de días de uso de ventilador mecánico de cada uno de los pacientes estudiados) lo que representa una incidencia acumulada de 44,92 casos por cada 1000 días de uso ventilador mecánico (muy por encima del valor nacional reportado por el MINSA). Las bacterias aisladas más frecuentes fueron *Acinetobacter baumannii* con 117 casos (35,5%), *Pseudomona aeruginosa* con 76 casos (22,4%), *Klebsiella pneumoniae* con 49 casos (14,5%) y *Staphylococcus aureus* con 46 casos (13,6%). El 15,0% restante se distribuyó entre los demás gérmenes (Tabla 1).

**Tabla 1:** Distribución absoluta y porcentual de los gérmenes de las neumonías asociadas a ventilador mecánico en la unidad de cuidados intensivos del hospital de alta complejidad «Virgen de La Puerta» de La Libertad.

Germen aislado	n	%
*Acinetobacter baumannii complex*	117	34,5
*Pseudomona aeruginosa*	76	22,4
*Klebsiella pneumoniae*	49	14,5
*Staphylococcus aureus*	46	13,6
*Stenotrophomonas maltophilia*	13	3,8
*Escherichia coli*	10	2,9
*Enterobacter cloacae complex*	10	2,9
*Enterobacter aerogenes*	7	2,1
*Serratia marcescens*	4	1,2
*Sphingomonas paucimobilis*	2	0,6
*Citrobacter freundii*	1	0,3
*Klebsiella oxytoca*	1	0,3
*Pseudomonas fluorescens*	1	0,3
*Proteus mirabilis*	1	0,3
*Pseudomonas putida*	1	0,3
Total	339	100,0

Fuente: datos obtenidos de los antibiogramas de los pacientes.

Los resultados de sensibilidad antimicrobiana difieren según el tipo de especie bacteriana aislada; para *Acinetobacter baumannii* se observó muy baja sensibilidad para carbapenémicos (10,2% para meropenem y 13,0% para imipenem), quinolonas (10,4% para ciprofloxacina y 13,5% para levofloxacino) y cefalosporinas (0,0% para ceftriaxona, 13,1% para cefepime y 14,0% para ceftazidima), manteniendo sensibilidad superior al 80,0% a colistina y tigeciclina (85,9% para tigeciclina y 100,0% para colistina); lo mismo sucede con *Pseudomona aeruginosa* que presentó una muy baja sensibilidad a carbapenémicos (30,6% para meropenem y 34,7% para imipenem), quinolonas (50,0% para ciprofloxacina y 50,0% para levofloxacina), cefalosporinas (0,0% para ceftriaxona, 48,1% para cefepime y 54,5% para ceftazidima), aminoglucósidos (68,9% para amikacina, 52,8% para gentamicina) y piperacilina/tazobactam (44,9%) manteniendo sensibilidad a colistina (100,0%). Para *Klebsiella pneumoniae* la sensibilidad muestra un mejor perfil para carbapenémicos (96,7% para meropenem y 98,0% para imipenem), quinolonas (72,9% para ciprofloxacina y 81,2% para levofloxacina), cefalosporinas (93,9% para cefepime y 90,6% para ceftazidima), aminoglucósidos (98,0% para amikacina y 88,0% para gentamicina) y piperacilina/tazobactam (96,9%). Para *Staphylococcus aureus* el perfil de sensibilidad de 100,0% fue para daptomicina, rifampicina, teicoplasmina y tigeciclina; lamentablemente no se tiene datos de oxacilina y vancomicina.

Según los resultados del presente estudio, es muy probable que la pandemia de la COVID-19 haya cambiado la frecuencia de gérmenes causantes de la NAV. Por ejemplo, el *Acinetobacter baumannii* ocupó el primer lugar, con 117 casos (34,5%); esto concuerda con lo reportado por el hospital Nacional Cayetano Heredia (HNCH) ^1^ que para el 2020 también reportó a este germen como el principal causante de las NAV, aunque con un porcentaje más alto (53,0%).

Con relación a la *Pseudomona aeruginosa*, esta presentó una baja sensibilidad a las cefalosporinas, quinolonas, amikacina y carbapenémicos. Estos datos muestran un perfil de sensibilidad no favorable y son similares a lo reportado por el HNCH, donde se menciona un 40,0% a 50,0% de sensibilidad para los carbapenémicos ^1^.

El presente estudio describe las NAV ocurridas en la pandemia de la COVID-19 y al compararlo con un estudio similar en UCI europeas, posterior a la segunda y tercera ola ^6^^,^^7^ coincide que los gérmenes más frecuentemente aislados son *Pseudomona aeruginosa*, *Escherichia coli*, *Klebsiella pneumoniae* y *Acinetobacter* spp., y cocos grampositivos, como *Staphylococcus aureus*.

Como limitaciones del estudio se debe reconocer que las muestras para el cultivo se tomaron a partir de secreción bronquial al momento de la aspiración y no de un lavado bronqueo alveolar y que en los resultados de los antibiogramas para *Staphylococcus aureus* no se contó con datos de oxacilina y vancomicina, por falta de insumo.

Finalmente, es muy posible que una de las causas del aumento de las NAV con resistencia bacteriana asociada fue el uso indiscriminado de antimicrobianos en la pandemia de la COVID-19 ^8^, en tal sentido el Centers for Disease Control and Prevention (CDC) informó un aumento de las infecciones con gérmenes resistentes, en especial de *Acinetobacter* resistente a los carbapenem, que aumento 78% en las infecciones; *Pseudomonas aeruginosa* multirresistente que aumento 32% en las infecciones, *Enterococcus* resistente a la vancomicina que aumento 14% en las infecciones y *Staphylococcus aureus* resistente a la meticilina que aumento 13% en las infecciones ^9^. Debido a esto se afirmó que la pandemia de la COVID-19 «hizo retroceder a los EE. UU. en su lucha contra la resistencia bacteriana» ^(^^9^. Asimismo, el uso de guantes por el personal de salud (que afectó la frecuencia de lavados de manos) pudo haber influido en el aumento de las infecciones resistentes. Afortunadamente, esta práctica fue desestimada en su totalidad en el 2022, por lo que esperamos que los resultados de futuros estudios sean diferentes.

En conclusión, la incidencia acumulada de la NAV en la UCI del hospital «Virgen de La Puerta» fue de 44,92 casos por cada 1000 días de uso ventilador mecánico y que debido a la pandemia de la COVID-19 hubo un cambio en el perfil bacteriológico y de resistencia de dichas infecciones, predominando la infección por gérmenes con elevada resistencia antibacteriana.
